# Monte Carlo simulations of synchrotron X-ray dose affecting root growth during in vivo tomographic imaging

**DOI:** 10.1038/s41598-023-32540-5

**Published:** 2023-04-06

**Authors:** Isabela C. Moraes, Dean Hesterberg, Fernando A. Bacchim Neto, Nathaly L. Archilha, Carlos A. Pérez, Maria Victória A. Araújo, Talita R. Ferreira

**Affiliations:** 1 Brazilian Center for Research in Energy and Materials (CNPEM), Campinas, Sao Paulo, 13083-970 Brazil; 2 Brazilian Synchrotron Light Laboratory (LNLS), Brazilian Center for Research in Energy and Materials (CNPEM), Campinas, Sao Paulo, 13083-970 Brazil; 3 Department of Biology, Federal University of Ceará (UFC), Fortaleza, Ceará, 60440-900 Brazil

**Keywords:** Abiotic, Atomic and molecular interactions with photons

## Abstract

Synchrotron X-ray computed tomography (XCT) has been increasingly applied to study the in vivo dynamics of root growth and rhizosphere processes. However, minimizing radiation-induced damage to root growth warrants further investigation. Our objective was to develop a robust approach for modeling and evaluating ways to reduce synchrotron X-ray dose effects on root growth during in vivo imaging. Wheat roots growing in soil were exposed to X-rays during XCT experiments resolved in space (3D) plus time (4D). The dose rate and cumulative absorbed dose in roots were modelled using the Monte Carlo code FLUKA for different experimental conditions of polychromatic and quasi-monochromatic X-ray beam configurations. The most impactful factors affecting damage to roots were incident X-ray energy spectrum, stored current in the accelerator machine, position of the root in the soil, and possibly the number of exposures during the 4D XCT experiments. Our results imply that radiation dose during in vivo imaging of plant roots can be diminished by using monochromatic radiation at the highest energy suitable for a given sample thickness and field of view, and by controlling the rotation axis of off-centered roots to increase attenuation of radiation by the soil matrix.

## Introduction

The development of X-ray computed tomography (XCT) in the late 1980s led to unprecedent ways of visualizing and quantifying the rhizosphere architecture in 3D^1^. Subsequently, great interest arose in XCT imaging of root development over time (4D imaging). This technique involves repeated imaging of a living root growing in soil or other substrate, typically over hours to days^2–5^. Such sequential imaging, however, can potentially attenuate root growth by radiation-induced damage from the deposited energy or dose. In fact, a number of investigations have focused specifically on characterizing the radiation dose delivered to roots during XCT analyses for various setups, especially for benchtop, medical, or industrial systems^1,3,6,7^.

Mooney et al.^1^ recognized more than a decade ago the importance of determining the effects of repeated exposures of plants to X-rays, and Zappala et al.^6^ reviewed the effects of X-ray dose in rhizosphere studies using XCT. These authors observed that most published studies on XCT had not reported or had provided insufficient information to calculate X-ray dose. Nevertheless, they created a database from 126 publications that allowed assessments of doses to samples using the Rad Pro X-ray Dose Calculator (http://www.radprocalculator.com/RadProDownloads.aspx). This calculator uses several approximations that are discussed by Lippold et al.^7^. Most of the calculated radiation doses in the database evaluated by Zappala et al.^6^ were lower than 33 Gy, which was considered at that time to be a threshold for significant detrimental effects on plant growth^8^. However, some studies involved doses as high as 10 kGy.

More recently, Blaser et al.^3^ reinforced that information on the influence of X-ray radiation on root growth was still scarce. They compared effects of cumulative X-ray dose on root growth of two plant species that were scanned every 2 or 4 days during a period of 17 days and reported a maximum cumulative dose < 8 Gy estimated using the Rad Pro X-Ray Dose Calculator. From measurements of several root parameters such as total root length, number, and length of second-order laterals, and length of first order laterals, they found that the two plant species differed in susceptibility to X-ray dose. Although Blaser et al.^3^ used the Rad Pro X-Ray Dose Calculator to estimate the cumulative dose in the root, they recognized some limitations of this approach. For instance, the calculator only accounts for radiation attenuation in air, completely disregarding the attenuation within soil. Also, input parameters of voltage, current, distance between source and sample, and filter material and thickness apply more to benchtop, industrial, or medical XCT systems than to synchrotron-based XCT that has become widely used in rhizosphere related investigations^9–12^. Later, Lippold et al.^7^ also showed that the Rad Pro Calculator substantially underestimated the radiation dose measured directly using dosimeters.

Scattering of photons and secondary electrons produced by the interaction of electromagnetic radiation with matter are important in evaluating the energy deposited in a root. Unlike the Rad Pro Calculator, the general-purpose Monte Carlo code, FLUKA^13^, accounts for the whole radiation-interaction cascade. FLUKA evaluates radiation interaction and transport for a variety of particles across an energy range between 100 eV (soft X-rays) to teravolts (cosmic rays) for primary radiation, and from kilovolts to teravolts for secondary electrons. In essence, this code provides highly accurate evaluation of macroscopic quantities such as energy deposition due to radiation interactions on a microscopic level. Therefore, it connects different physics-based models to build a powerful tool that can be used to validate experimental data and to make predictions when no experimental data are available.

With advances in technology to produce and detect X-rays, image-based studies of rhizosphere processes are in the spotlight (e.g., Schnepf et al.^14^). The advent of 4th generation synchrotron light sources provides X-ray beams of high photon flux^15^ and enables very fast (seconds) XCT scanning for temporal studies. For example, the MOGNO beamline at the Sirius synchrotron in the Brazilian Synchrotron Light Laboratory (LNLS)^16–18^ is designed to produce fast tomographic images (≥ 1 XCT/s) in a zoom tomography configuration using quasi-monochromatic radiation maximized at one of three selected energies (https://www.lnls.cnpem.br/facilities/mogno-en/). This range of characteristics provides capabilities to follow fast, dynamical processes in the rhizosphere over time and at multiple spatial scales^16,19^. And it also gives flexibility to select data-collection parameters for acquiring high-resolution in vivo XCT data while minimizing radiation-induced damage to the roots.

The dose modeling work reported here was initiated in response to observations of rapidly diminishing root growth during in vivo measurements made during a first phase of the MOGNO beamline development (referred herein as MOGNO-A). MOGNO-A used a polychromatic, filtered beam from a 3.2 T Central Bending Magnet (BC) source without optical focusing. The final beamline design, MOGNO-B, uses multi-layer coated mirrors to produce quasi-monochromatic radiation with maximum photon flux at 22, 39, or 67.5 keV energies, and the zoom configuration provides fields of view (FOVs) ranging from 85 mm to 150 µm with corresponding spatial resolutions of 55 µm to 120 nm. The specific objectives of this study were to develop a robust X-ray dose model that explained apparent radiation effects on root growth during in vivo imaging at MOGNO-A, and to utilize this model to optimize imaging parameters for MOGNO-B that are favorable for 4D tomography with minimal radiation-induced damage to root growth. The FLUKA-based dose model presented here considers variables that were not accounted for in previous works, including the entire energy profile of the X-ray beam, soil composition, and varying path length of the beam through soil during an XCT scan. We hypothesized that there are optimal combinations of parameters (energy, FOV, and sample configuration) that can be controlled at MOGNO-B to obtain high-contrast, in vivo images of roots growing in soil with acceptable levels of radiation dose to minimize damage to growth.

## Material and methods

### 4D XCT experiments at MOGNO-A

Detailed procedures on soil preparation and wheat seedling germination and growth in pipet tips are presented in the Supplementary Information 1-Method 1, Figure S1. All methods were performed in accordance with the relevant institutional, national, and international guidelines and legislation.

**Figure 1:**
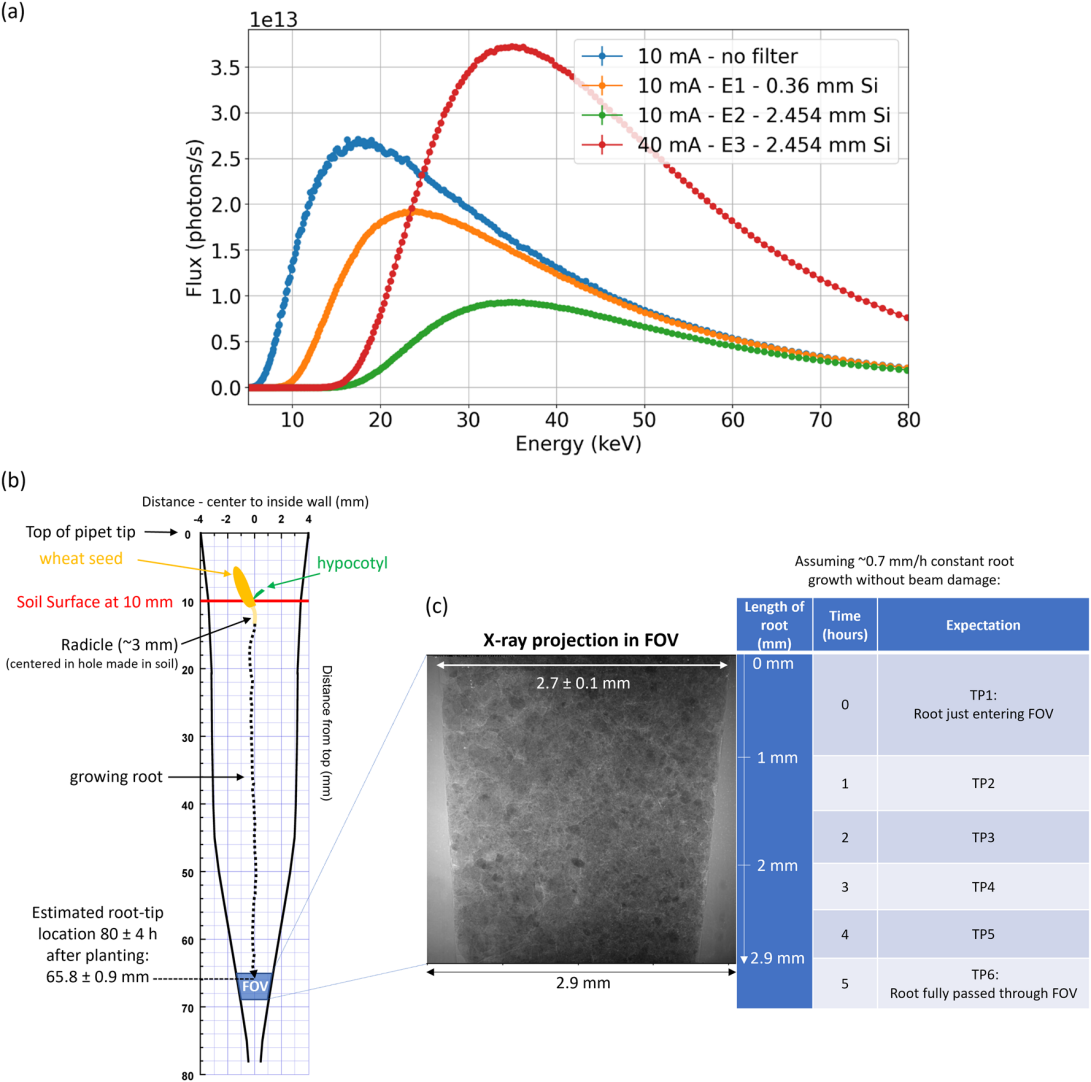
(**a**) Polychromatic X-ray beam spectra from the central bending magnet (BC) source for different silicon (Si) filter thicknesses and Sirius storage-ring currents used in experiments E1, E2, and E3 at MOGNO-A. Schematics showing (**b**) mapped length and internal diameter of a 100 – 1000 µL pipet tip with the estimated position (± standard deviation) of a wheat root grown for ~ 80 h relative to the optimal field of view (FOV) constrained by the detector system; (**c**) typical X-ray projection collected across the FOV and table exemplifying the expected root position in the FOV at different time points (TP) assuming the root tip is positioned 0.1 – 1 mm from the top of the FOV at TP1.

Three XCT experiments were performed with varying beam configuration parameters as specified in Table 1. Because this study was performed during the commissioning of the Sirius accelerator, the machine operating current was 10 mA for experiments 1 (E1) and 2 (E2), and 40 mA for experiment 3 (E3) (Table 1). Also, the experiments were conducted during the commissioning of a preliminary configuration of the MOGNO beamline (MOGNO-A) receiving polychromatic beam (energy peak, E_p_, ~ 18 keV and mean energy, E_m_, ~ 25 keV) coming directly from the BC source without optical focusing (Fig. 1a). Silicon filters of varying thicknesses were used to harden the beam such that E_p_ and E_m_ were ~ 23 keV and ~ 31 keV in E1, and ~ 35 keV and ~ 42 keV in E2 and E3 (Fig. 1a, Table 1). The time per projection in each experiment was set targeting approximately 35,000 photon counts (about half of the photon counting needed to saturate the detector). This criterion for the acquisition time ensured a satisfactory signal-to-noise level in the reconstructed images. Typically, the acquisition time increases with increasing filter thickness and decreases with increasing stored ring current. All images were reconstructed by applying a filtered back projection-based algorithm^20^. The reconstructed images were 16-bit, hdf5 type, with a maximum array of 1,024 × 1,024 × 1,024 voxels, and a 2.88 μm voxel size, equivalent to the geometrical resolution (Supplementary Information 1-Method 2).

**Table 1:** XCT data collection parameters used for different experiments (E) and imaged roots (R).

	E1R1	E2R1	E2R2	E3R1/E3R2
Number of image projections	256	256	1024	512
Stored ring current (mA)	10	10	10	40
Si filter thickness (µm)	360	2450	2450	2450
Image X-ray transmission (%)	~ 50	~ 70	~ 70	~ 70
Time per projection (s)-nominal	0.15	0.47	0.56	0.009
Time per projection (s)-average	0.16	0.48	0.57	0.015
Full acquisition time (s)	41.35	123	583.8	7.68

^†^Areal detector system: PCO.edge 4.2; Scintillator: LuAg:Ce; Physical detector pixel size: 7.2 µm; Original number of pixels per column and row: 2048; Objective: 5x; Camera binning: 2 × 2; Resulting number of pixels per column and row: 1024; Effective pixel size: 2.88 µm × 2.88 µm; Rotation angle: 180°; FOV: 2.9 mm × 2.9 mm.

In each experiment, a first XCT image (TP1) was made until a candidate sample was found with 0.1 to 1 mm of root tip entering the top of the 2.9-mm FOV (Figs. 1b,c). The projected times for a root tip to enter the FOV (Fig. 1b,c) were based on growth rates measured in preliminary experiments (Supplementary Information 1—Figure S2) based on the time after planting for roots to emerge from the bottom outlet of pipet tips. Ideally the root would be centered, but most roots imaged were growing closer to the pipet-tip wall. This initial screening was needed because of imprecision in predicting the location of the root based on measurements of root/shoot length ratios of wheat determined from preliminary experiments. Candidate samples were either left in the XCT stage (E1 and E3) or removed and returned to the stage at the same coordinates (E2) for collecting a series of XCT images at ≥ 1-h intervals. If there was no beam damage, the root should grow through the FOV in ~ 4 h (Fig. 1c).

For Experiment 2, at 7.4 and 21 h after TP1, when the first root that entered the FOV (E2R1) stopped growing, higher-quality images were also made using a greater number of projections and longer exposure times to achieve higher signal-to-noise ratio. These images corresponded with TP1 and TP2 of a second root (E2R2) that had entered the FOV (Table 1; Fig. 4d,e). Note that we use the terminology “E#R#”, where E# stands for “Experiment number” and R# represents “Root number” in chronological order of entering the tomographic FOV in a given experiment (e.g., E1R1 means experiment 1 and first root in this experiment entering the field of view).

The root growth was measured along each experiment based on the reconstructed (grayscale) images. The growth step was determined by counting the incremental number of voxels containing the root between TPs and multiplying by the voxel size (e.g., Fig. 4a). The root growth for TP1 was determined from the length of the root grown in the pipette tip between the times of planting and the first XCT scan (see Fig. 1b). Because different time intervals were used between each TP among experiments, root growth was analyzed in terms of growth rate (mm/h). Only the image collected at TP2 for E2R2 was segmented to visualize pore space, solid matrix, roots and root xylem.

### FLUKA modelling of radiation dose to roots

X-ray dose calculations were performed using the FLUKA Monte Carlo method via Flair, its graphical user interface for inputs and results visualization (version 3.1–15)^21^. The FLUKA base code uses a statistical approach for radiation interaction and transport through matter, and our inputs included beam and sample geometry and a number of physical parameters. The calculations for this study were developed using the clusters SDUMONT/LNCC and CENAPAD/UNICAMP for a higher computational performance to achieve a maximum of 10% statistical uncertainty in a short period (1–30 h).

#### Geometry of the model system

**Figure 2:**
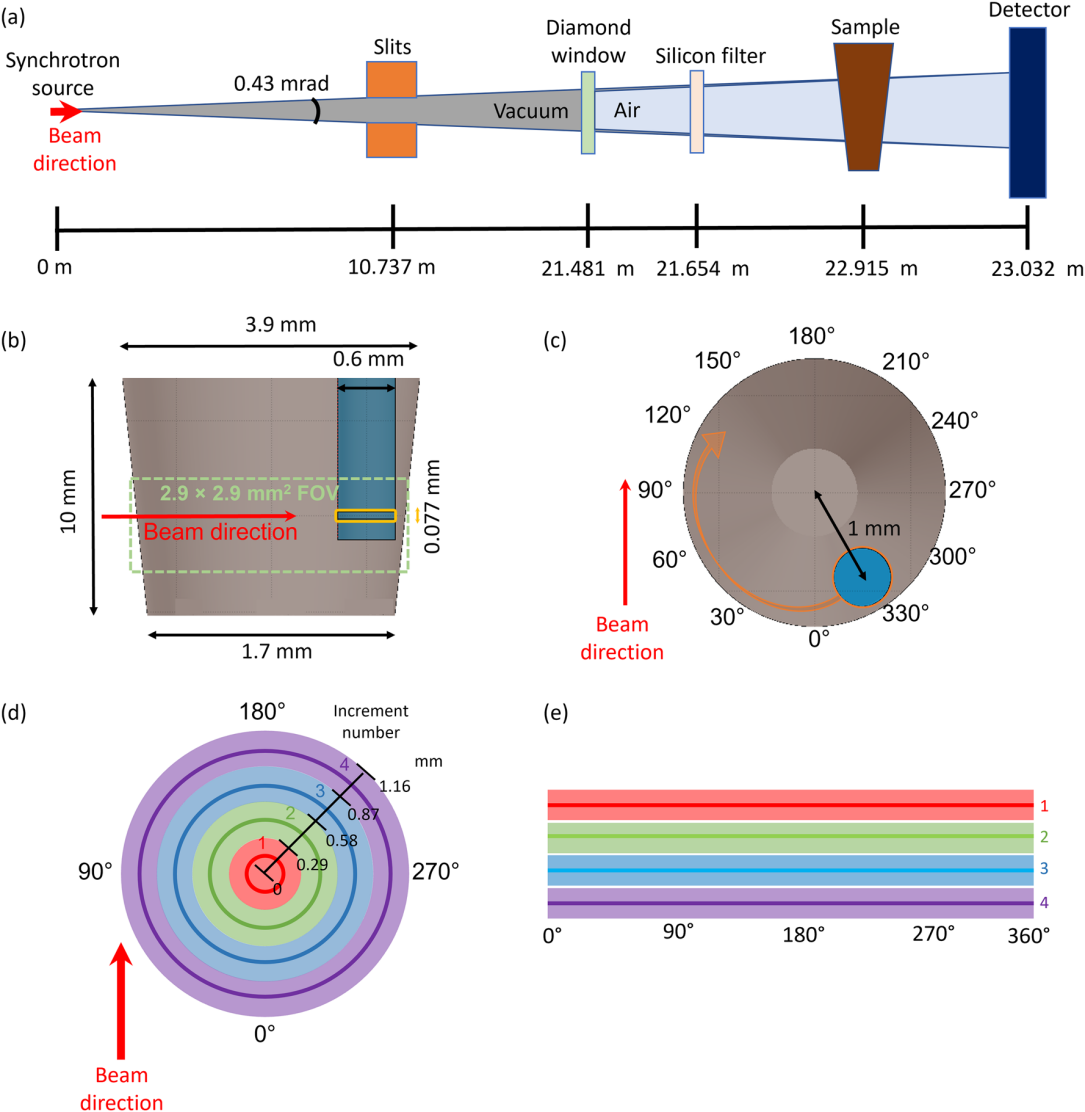
(**a**) Schematics of MOGNO-A beam configuration geometry implemented in computational simulations. Schematics of FLUKA geometry in a (**b**) longitudinal section, where the dashed green box represents the 2.9 × 2.9 mm^2^ FOV and the yellow box represents the segment of the root that was illuminated by the beam in the simulations—figure not to scale, and a (**c**) cross section. Cylindrical coordinate representation in FLUKA detector analyses: (**d**) concentric soil cylinder volume increments had 0.29 mm of thickness and (**e**) corresponding region as a function of angle for each increment.

The simulations followed the actual design of XCT experiments for the MOGNO-A beamline configuration. Simulations of the MOGNO-A geometry (Fig. 2a) used the BC source dimensions of 22.1 × 8.5 µm rms with an angular divergence of 0.43 mrad (limited by slits) for the X-ray beam. This geometry resulted in a FOV of 2.9 × 2.9 mm^2^ on a sample placed 22.915 m from the BC source. The beam travelled inside a vacuum chamber for 21.481 m, then through a 0.2 mm thick diamond window. The Si filter used in the experiment was modelled as a 10 × 10 cm^2^ rectangular plate in air at 21.654 m from the source, and its thickness was varied (Table 1). A 120 mm aluminum box, representing the areal photon detector on the beamline, was positioned at a fixed distance of 23.032 m from the BC source and was included to account for any backscattered photons.

The soil composition implemented in the simulations consisted of a mixture of nine elements (Supplementary Information 1—Table S1), with particle density estimated as 2.7 g/cm^3^. Based on the segmented E2R2-TP2 image, the initial soil sample porosity was 30% (v/v), which was arbitrarily assumed in the simulations to be equally divided between water (1 g/cm^3^) and air (0.001225 g/cm^3^) (i.e., 15% v/v each). Therefore, the overall sample density was determined as the weighted mean of the three component densities, resulting in 2.04 g/cm^3^. The root was assumed to be composed of only water (1 g/cm^3^).

The soil geometry implemented in the simulations (Fig. 2b) reproduced that of the FOV of the soil-filled pipette tip (Figs. 1b,c). Each root was implemented as a water filled cylinder, with 0.6 mm diameter and 5.25 mm height, to include eventual radiation scatterings around the root vicinity. A segment of the root defined by a parallelepiped of 0.077 mm × 0.077 mm squared face and 0.6 mm length along the axis of the beam was fully illuminated by the beam (orthogonal cross section view in Fig. 2b) in the simulations. The absorbed dose is given in energy per mass (J/kg or Gy); thus, analysis of the chosen segment can be scaled to the root as a whole. The center of the model root was positioned at a 1 mm radial distance from the central symmetry axis of the conical soil and was rotated around 180° for simulations (Fig. 2c). The geometry considered in all calculations to model the experiments at MOGNO-A was the same, i.e., root growth (in diameter, length, or volume) over time was not considered. A FLUKA input text for the scenario shown in Fig. 2c is given in Supplementary Information 2.

The energy of photons in each code initialization was set using a FLUKA source routine to read text files containing the synchrotron X-ray beam profile (flux as function of energy) between 5 and 400 keV, corresponding to the total flux of $4.61 \times {10}^{13}$ photons/s/mA (Fig. 1a, no filter curve). The number of photons in the initialization of the simulations (called primaries in the code) was on the order of ${10}^{9}$ to achieve an uncertainty below 10%. The energy threshold was established with an EMFCUT card to produce and transport photons and electrons above 100 eV and 1 keV, respectively^13^.

For all dose analyses, USRBIN detectors were applied with a ‘DOSE' parameter for absorbed dose quantity (GeV/g/primary). The output value was multiplied by $1.602176462 \times {10}^{-7}$ for conversion to Gy/primary, then multiplied by the beam flux for each spectrum, to obtain absorbed dose rate in Gy/s/mA.

#### Dose estimation in a root segment

The model simulated a root and soil rotated around its central symmetry axis (Fig. 2c), similar to collection of XCT data at MOGNO-A for each TP (Table 1). The absorbed dose rate in the root segment (highlighted in yellow in Fig. 2b) was evaluated at seven angular increments of 30° along a clockwise rotation of 180°, similarly to experiments, as represented in Fig. 2c. The starting position of the root for each simulation was based on that of the physical experiment (Fig. 4a-discussed below). Since the dose rate distribution is symmetric, it was graphically conveyed with an angle variation over a full rotation (360°) of the root.

The parameter ‘Region’ was used in the USRBIN detector to evaluate the dose rate in the root segment. For this parameter, as required by the code algorithm, the result needs to be divided by the volume of the exposed root segment, i.e., $2.93 \times {10}^{-6}$ cm^3^.

To determine the cumulative dose at each TP, the dose rate as a function of the angle of the root segment was fitted using a polynomial function and integrated between the starting angle relative to the beam vector and the angle after a clockwise rotation of 180°, then multiplied by the stored ring current and the XCT full acquisition time (Table 1). An uncertainty of 10% was considered for each value resulting from the integration because this uncertainty is the associated maximum in each simulation. For different TPs of a given experiment, the starting position of the root was fixed. Therefore, the cumulative dose calculated for one TP was multiplied by the number of TP measurements to obtain the total cumulative dose for each experiment.

#### Spatial dose distribution in soil

To evaluate the impact of soil attenuation, a spatial dose rate map in soil was generated using volumetric detectors with cylindrical coordinates (“R-Φ-Z” parameter). Three-dimension volume binning was used to visualize the dose rate maps. The voxel size was 0.0025 mm, and the angular step was one degree. The dose rate simulation in each detector voxel was performed as a function of angle for four concentric increments of soil cylinder volumes (Fig. 2d). Note that the root was not included because this analysis was intended to evaluate the dose-rate trend over the soil cross section, which would be similar in the presence of a root. By averaging the results over each incremental soil cylinder volume, radial curves were generated and translated to horizontal lines in cylindrical coordinates (Fig. 2e). An angular path that minimizes the cumulative dose was determined by integrating these curves, analogous to that described in the previous section.

#### Minimizing dose with the MOGNO-B beamline configuration

**Figure 3:**
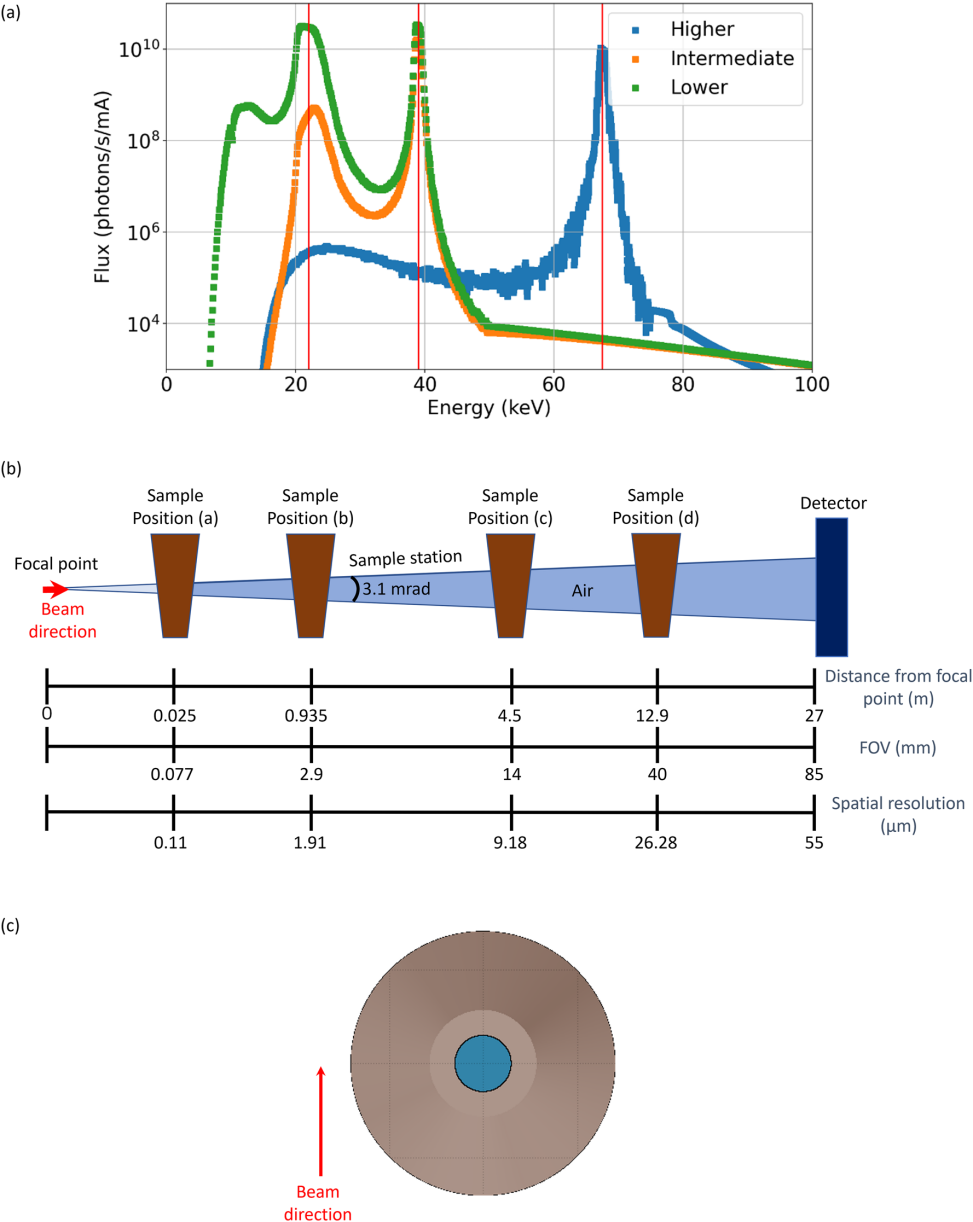
(**a**) Quasi-monochromatic beam spectra of the MOGNO-B configuration after optical focusing and energy selection by KB mirrors, with a 0.7 mm (lower energy) or 7.07 mm (intermediate and higher energies) silicon filter included. (**b**) Schematics of the MOGNO-B beam configuration for four example positions of a soil sample (figure not to scale). (**c**) Schematics of the FLUKA geometry for a soil cross section with a root in the central symmetry axis, which was used for simulations to compare the MOGNO-A and -B configurations.

The final MOGNO beamline (MOGNO-B) was designed to work in a cone beam configuration (X-ray beam focus of 100 nm size) with energy discrimination in a direct photon detector to provide three selectable quasi-monochromatic energies of 22, 39, or 67.5 keV. Optical focusing and selection of beam-energy in MOGNO-B is provided by a set of elliptical, KB (Kirkpatrick-Baez) mirrors producing three quasi-monochromatic cone beam configurations with maximum fluxes at lower (22 keV ≅ 39 keV), intermediate (39 keV > 22 keV), and higher (67.5 keV) X-ray energies. For this work, we used the energy spectra impinging on the sample (Fig. 3a) after the mirrors and either a 0.71- or 7.07-mm thick silicon filter. Including Si filters in modeling of MOGNO-B had a negligible effect on the more prominent energy peaks of the beam profile delivered by the KB mirrors, but attenuated X-rays of lower energies.

A quantitative comparison of dose rate on a sample diameter of 2.9 mm was performed between a polychromatic beam with 0.36- and 2.45-mm thick Si filters at MOGNO-A (experimental configurations used here, Fig. 1a; Table 1) and the three quasi-monochromatic beam spectra of lower, intermediate, and higher energies at MOGNO-B (Fig. 3a) to evaluate which beamline parameters and experimental setups would diminish dose during in vivo XCT imaging of a root. At MOGNO-A, only one FOV (2.9 mm) was possible (Figs. 1b,c). At MOGNO-B, the X-ray cone beam with 3.1 mrad angular divergence provides varying FOVs and spatial resolutions depending on the distance between the X-ray beam focus and the sample. (Supplementary Information 1 -Method 2, Eq. S1). The four sample positions (distances) and their corresponding FOVs and spatial resolutions included in our simulations for three different sample sizes are specified in Fig. 3b, and the detector was fixed at 27 m from the beam focus. The 2.9 mm thick sample was modeled at MOGNO-B both at 0.025- and 0.935-m distances from the focus (positions a and b in Fig. 3b). The 0.025-m source-to-sample distance provides a 0.077-mm FOV and 0.11-µm spatial resolution, and the 0.935-m distance provides a FOV of 2.9 mm (Supplementary Information 3), equal to that of MOGNO-A, and a spatial resolution of 1.91 µm.

Additional dose rate simulations were performed for 14- and 40-mm thick soil samples in FOVs of equivalent sizes at positions c and d in the MOGNO-B cone beam (Fig. 3b). The simulations used the intermediate- and higher-energy spectra (Fig. 3a) for the 14- and 40-mm samples, respectively, targeting an optimal X-ray transmission of ~ 30%. This optimal transmission is an approximation based on empirical observations and is justified by being close to the condition in which the sample thickness equals the calculated absorption length of the soil material. The smallest FOV of 0.077 mm (position a, Fig. 3b) was also simulated for these thicker samples. From a biological perspective, the intermediate- and higher-energy spectra are particularly important for measuring sample thicknesses that include a larger volume of roots and associated rhizosphere. The same conical soil and sample geometry was used for all simulations as described previously, except the root was centered (Fig. 3c) for those simulations. The 3D FLUKA detector with a Cartesian coordinate system (X–Y-Z) used cubic voxels of 0.0025 mm.

For the quasi-monochromatic beam simulations, the sampled spectra considered all photons reaching the sample holder (from 1 to 100 keV, with a flux of $6.01 \times {10}^{9}$, $5.01 \times {10}^{8}$ and $1.47 \times {10}^{8}$ photons/s/mA for lower, intermediate, and higher energy setups, respectively; Fig. 3a). The lower X-ray flux simulated for MOGNO-B relative to that of MOGNO-A is due to loss of flux on the mirrors and through the silicon filters.

## Results

### Simulation of experimental data from MOGNO-A

#### In vivo* root growth*

**Figure 4:**
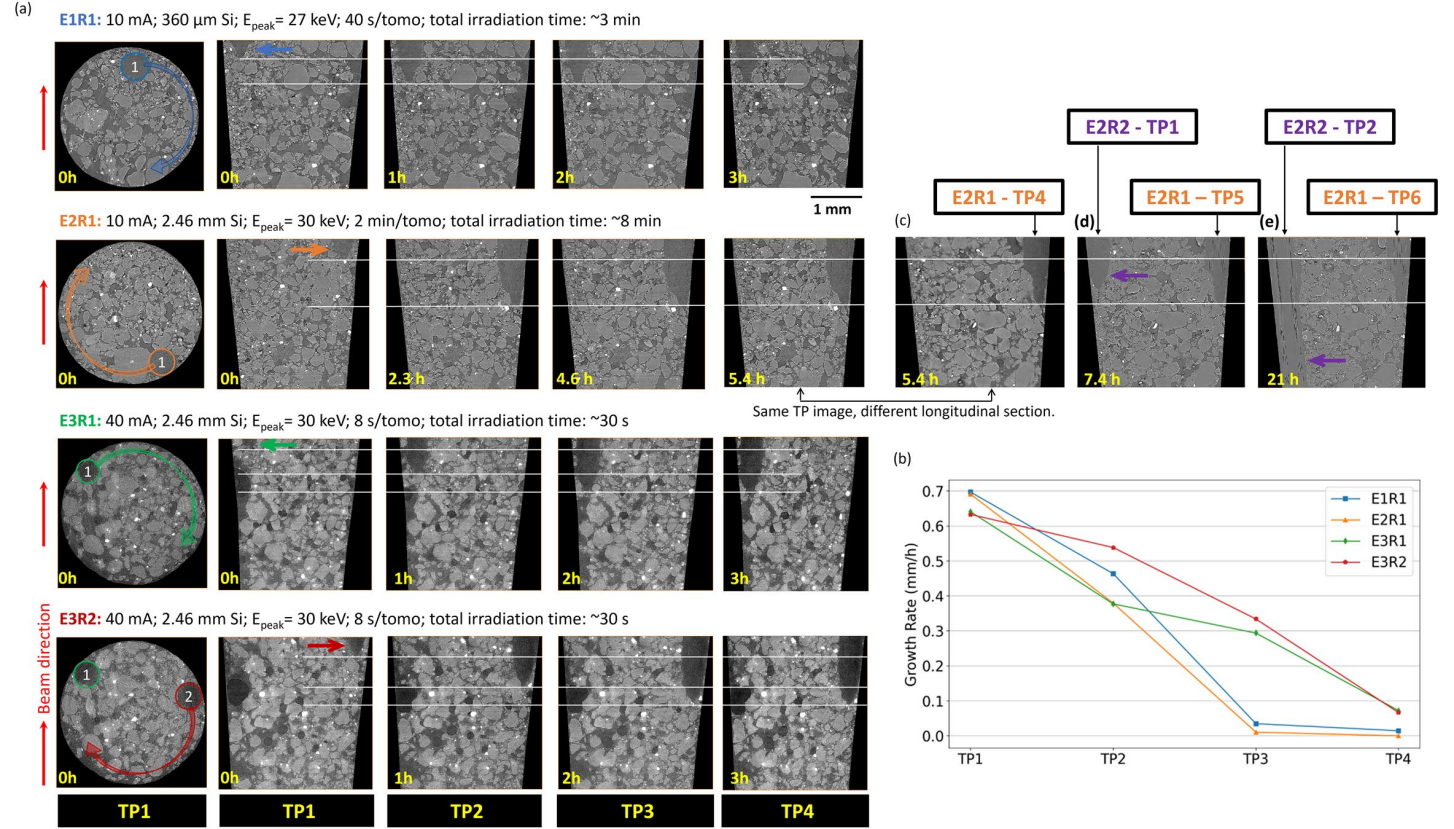
(**a**) Top and longitudinal cross sections of each studied root taken at different time points (TPs). Arrows in the top cross sections indicate the range of sample rotation, and horizontal lines in the longitudinal sections denote the different positions of the root tip across TPs. (**b**) Root growth rate between TPs for each experiment. Longitudinal sections at (**c**) E2R1—TP4 – showing the final growth of the first root that entered the FOV, (**d**) E2R2—TP1 – showing a second root that entered the FOV after the first root stopped growing, (**e**) E2R2—TP2 – showing the second root grown past FOV. The longitudinal section was changed from Fig. 1a to 1c,d,e to allow simultaneous visualization of roots E2R1 and E2R2. E—experiment; R—root; TP—timepoint; FOV—field of view (see the video in Supplementary Information 4 for 3D rendering of a segmented image of Fig. 1e).

Figure 4a shows a cross section from the top of the FOV in each experiment, where circles indicate the starting root positions relative to the X-ray beam direction, and the associated arrow indicates the range of angles at which the root was imaged during 180° of sample rotation to collect tomographs. The roots from the different experiments had diameters of 490 ± 50 µm in their most mature parts captured in the imaging FOV (Fig. 4a). In addition, images for longitudinal sections collected at four TPs, (e.g., 0, 1, 2, 3 h) for each experiment reveal the root growth and consequent changes in the solid particle arrangement around it. Whereas there was significant root growth only across the time interval between TP1 and TP2 for both E1R1 and E2R1, there was significant growth across two time intervals for E3R1 and E3R2 (Fig. 4a). These differences are summarized in Fig. 4b, which shows that root-growth rate diminished over sequential TPs for all experimental configurations, but less reduction was seen for the two roots in E3. An alternative version of the plot in Fig. 4b, showing growth rate as a function of time, is presented in the Supplementary Information 1 - Figure S3. In addition to the reference growth rate of 0.6–0.7 mm/h for roots to reach the FOV (TP1), results in Figure S2 (Supplementary Information 1) verified that in the absence of X-ray exposure, roots from 80% of the wheat plants continued to grow at overall rates of 0.5 to 0.8 mm/h from the depth of planting, through the FOV, and out the bottom of the pipet tips.

Growth of root E2R1 stopped after TP2, and TP4 occurred 5.4 h after TP1 of E2R1 (Fig. 4a,c). When a higher-quality (i.e., higher signal-to-noise ratio) image was taken (2 h later) with an acquisition time (583 s) more than 4.5-fold longer than other images (Table 1, E2R2 vs. others), a second root was imaged entering the FOV (E2R2—TP1: 7.4 h after E2R1—TP1; Fig. 4d). Another higher-quality image taken 13.6 h after E2R2—TP1, i.e., 21 h after the E2R1—TP1, showed that root E2R2 had crossed the entire FOV. Although we hypothesized that radiation damage would arrest the growth of E2R2 as well as E2R1, it was intriguing that E2R2 continued to grow despite the nearly fivefold longer time used to collect a high-quality image.

The video in Supplementary Information 4 shows a high-quality, segmented root-soil animation that could be acquired without, in the case of root E2R2, stopping root growth. Although the (not segmented) images shown in Fig. 4a were of sufficient quality to follow root growth over time as was done by Keyes et al.^11^, higher-quality images such as in Figs. 4d,e and in the video from Supplementary Information 4 would be better for in vivo studies of more detailed rhizosphere phenomenon such as root growth effects on soil particle movement and structure development,^10,11^ or growth in relation to the location of fertilizer granules^2,22^.

#### Dose rate and cumulative dose simulations for each experiment

**Figure 5:**
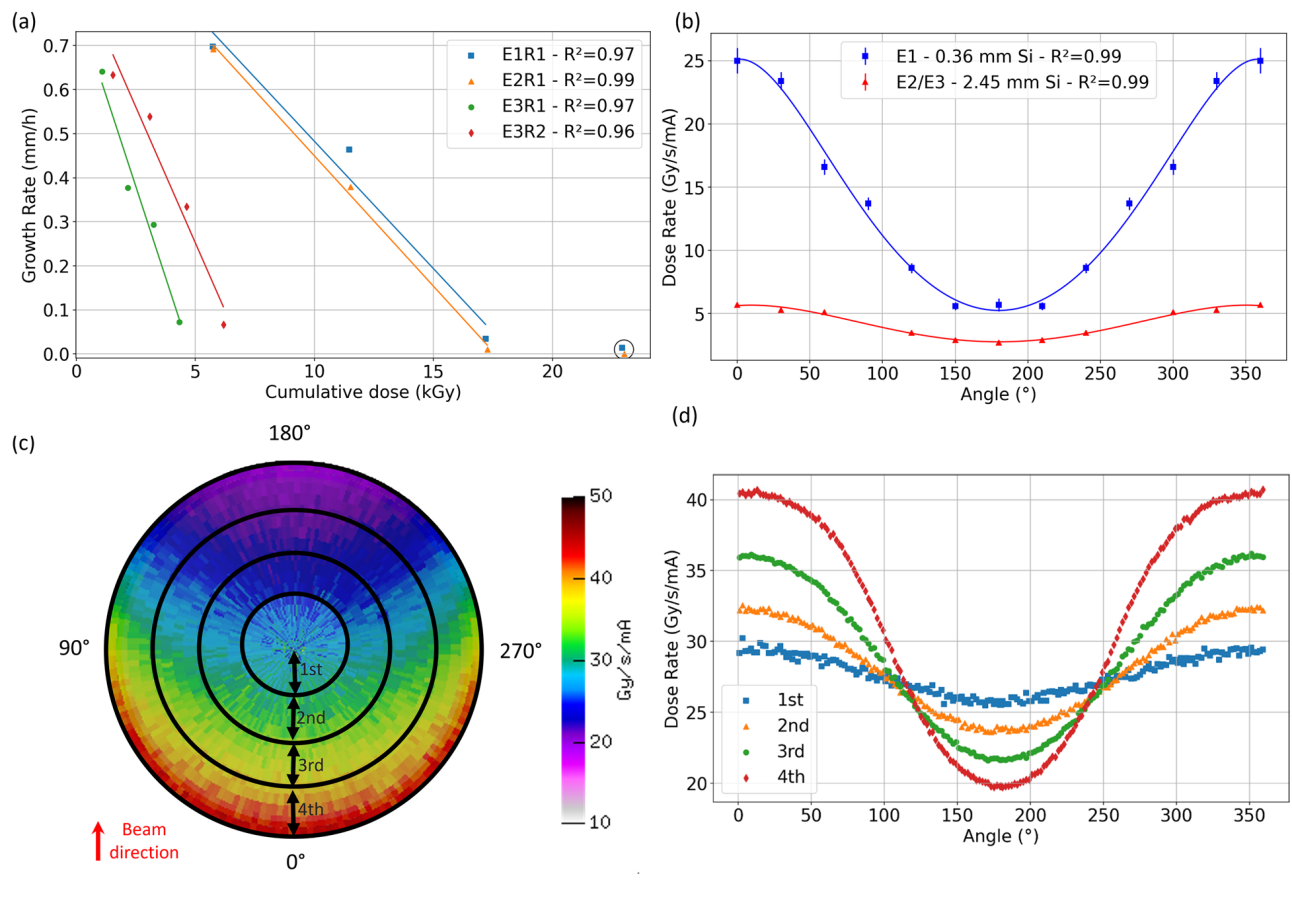
(**a**) Root growth rate as a function of modeled cumulative absorbed dose on the root for each experiment, where consecutive data points from left to right on each curve represent the sequence of timepoints (TP1, TP2, TP3, and TP4). The circled points were not included in the linear regressions because in these curves the roots had essentially stopped growing between TP2 and TP3. (**b**) Modeled effect of rotation angle and Si-filter thickness on the dose rate (normalized by current) on a root positioned along the pipet-tip wall, where 0° represents the angle between the vector of the incoming beam and a vector projected from the root to the center of the sample (see Fig. 2c). A 0.36 mm filter was used in E1, and a 2.45 mm filter was used in E2 and E3. (**c**) Modeled dose rate distributions across incremental cylindrical volumes of a soil sample irradiated with X-rays filtered through 2.45 mm thick Si at MOGNO-A. (**d**) Dose rate as a function of angular position for each soil-cylinder volume increment when using a 2.45 mm Si filter. E—experiment; TP—timepoint.

Figure 5a shows that the root growth rates decreased linearly with cumulative modeled dose. The slopes and positions of the curves were different because the experimental conditions varied between E1, E2, and E3, as discussed below. Roots E3R1 and E3R2 showed diminished growth rates at lower cumulative absorbed doses than roots E1R1 and E2R1. However, the absolute decline in growth rate between TPs was less for the roots in E3 than for those in E1 and E2, apparently because of the lower incremental changes in cumulative dose between TPs in E3.

For a root near the capillary wall, simulated radiation dose rates varied with Si filter thickness and the rotational position of the root relative to the incoming beam (Fig. 5b), where 0° represents a root directly in front of the beam (Fig. 2c). At 0 and 360°, the X-ray beam is impinging on the root without being attenuated by soil material, thus resulting in the greatest dose rate. The soil attenuation of the beam is maximal at 180° when the root is opposite the incoming beam. This angular dependence of dose rate varied by fivefold for the X-ray beam filtered through 0.36 mm of Si, which had a radiation profile skewed toward lower-energies (Fig. 1a), and it varied by more than twofold with angle for 2.45-mm filtered radiation. Essentially, the soil, which is largely composed of low-Z elements (nearly 90% oxygen, silicon, and aluminum—Supplementary Information 1—Table S1) more effectively attenuates lower energy X-rays that are also absorbed to a greater degree by the root.

Modeled X-ray dose rate distributions across incremental soil cylinders at various radial distances from the sample center (as defined in Figs. 2d,e) were affected by beam attenuation by the soil (Fig. 5c). Moreover, Fig. 5d shows curves corresponding to each incremental-cylinder averaged dose rate as a function of rotation angle (see Fig. 2c). These results indicate that the outer-most soil cylinder increment (4th layer) is subjected to a highest maximum and lowest minimum absorbed dose rate because this geometry gives the greatest variation in soil thickness across rotation angles. In addition, the integrated dose rate was lowest for the outer soil-cylinder increment for a clockwise rotation from 90° to 270° (4.4 kGy/s/mA) and highest for a rotation from 270° to 90° (7.0 kGy/s/mA) (Table 2). This difference is less than twofold, corroborating that root position and rotation angle are not the dominant contributors to the fivefold variation in incremental dose between E1/E2 and E3 (Table 3). Therefore, the beam profile characteristics, i.e., Si filtering and stored current likely play a more important role.

**Table 2:** Modeled absorbed dose rates for incremental soil cylinders at varying radial distances from the center at 0 mm and for varying rotation angles, using the MOGNO-A configuration with a 2.45 mm Si filter as illustrated in Figs. 2d and 5c.

	Rotation angle*	Radial increments from the center of a 2.9 mm diameter cylinder soil (mm)			
		0 – 0.29	0.29 – 0.58	0.58 – 0.87	0.87 – 1.16
Absorbed dose rate (kGy/s/mA)	0° $\leftrightarrow$ 180°	4.9 ± 0.5	5.0 ± 0.5	5.2 ± 0.5	5.5 ± 0.6
	45° $\to$ 225°	4.9 ± 0.5	4.8 ± 0.5	4.7 ± 0.5	4.9 ± 0.5
	90° $\to$ 270°	4.8 ± 0.5	4.6 ± 0.5	4.5 ± 0.4	4.4 ± 0.4
	225° $\to$ 45°	5.2 ± 0.5	5.5 ± 0.6	6.0 ± 0.6	6.7 ± 0.7
	270° $\to$ 90°	5.3 ± 0.5	5.7 ± 0.6	6.2 ± 0.6	7.0 ± 0.7

*Clockwise rotation.

**Table 3:** Modeling results showing incremental and cumulative absorbed doses by each root imaged at MOGNO-A.

	E1R1	E2R1	E2R2*	E3R1	E3R2
Si filter thickness (mm)	0.36	2.45	2.45	2.45	2.45
Stored ring current (mA)	10	10	10	40	40
Full acquisition time per TP (s)	41.35	123	583.8	7.68	7.68
Angle range in clockwise rotation (°)	180 → 0	330 → 150	150 → 330	135 → 315	240 → 60
Dose per XCT TP (kGy)	5.7 ± 0.6	5.8 ± 0.6	22 ± 2	1.1 ± 0.1	1.5 ± 0.2
Cumulative dose (kGy)—4 TPs	23 ± 2	23 ± 2	-	4.3 ± 0.4	6.2 ± 0.6

*This result was not considered for root growth analysis because exact growth rate measurements could not be made.

^†^The 4 TPs considered for the cumulative dose are those from Fig. 4a.

Over a rotation of 180°, E1R1 and E2R1 covered almost inverse paths symmetric to the beam direction (180 → 0 vs. 330 → 150; Table 3), which are expected to result in approximately the same dose rate, regardless of the filter thickness used (e.g., 0° ⟷ 180°; Table 2). However, although the increase in filter thickness from E1R1 to E2R1 has an associated two- to fivefold decrease in dose rate along these angular ranges (Fig. 5b), the acquisition time caused a threefold increase because the same stored current was used (Table 3). The acquisition time had to be increased in this case to compensate for diminished beam intensity (at the same stored current) caused by the thicker filter (Fig. 1a). The counter effects of increasing the Si thickness and increasing the acquisition time resulted in similar incremental (per TP) and cumulative dose (4 TPs) between E1R1 and E2R1 (Table 3).

On the other hand, although the dose rate was the same for E2 and E3 (Fig. 5b; 2.45 mm Si), the stored ring current was fourfold higher in the later (Fig. 1a) and the acquisition time, therefore, was lower (16-fold) (Table 3). Thus, the dose rate from Fig. 5b integrated in the experimental angular ranges multiplied by ring current and acquisition time resulted in fivefold lower incremental and cumulative absorbed doses for E3 compared with E2 (Table 3). In accordance, the experimental data from Fig. 4b indicated that between TP2 and TP3, X-rays exposed on E3R1 and E3R2 caused a lower reduction in the root growth rate than in E1R1 and E2R1. On the other hand, incorporating rotation angle into the dose model showed that the orientation relative to the beam of the root growing along the pipet tip wall (Fig. 4a) was a minor contributor to the incremental dose (Table 3) in E3. Angle effects could only be compared between E3R1 and E3R2, which used the same Si-filter thickness, stored current, and full acquisition time per XCT TP, with angle range being the only factor varying (Table 3). Indeed, considering the maximum twofold influence of the root position and rotation angle on the dose rates in Table 2, it appears that the beam profile (i.e., Si filtering) and acquisition time (strongly influenced by the stored current) are major contributors to the differences in incremental and cumulative doses observed among experiments (Table 3). Particularly, between E2 and E3, the reduction in acquisition time per TP due to the increase in stored current was the critical factor.

### Effects of polychromatic (MOGNO-A) vs. monochromatic X-ray beam configurations (MOGNO-B)

Our modeling results for the MOGNO-A and MOGNO-B beamline configurations predict that the energy spectrum of radiation used in XCT data collection impacts absorbed dose by up to four orders of magnitude (Table 4). Energy and FOV have the greatest effect on dose rate, with higher energy and larger field of view decreasing dose. For example, filtering to skew the radiation spectrum toward higher energies in the MOGNO-A configuration decreased modeled dose rate from 8,900 to 3,100 mGy/s/mA. Similarly, for the same 2.9-mm sample size and FOV at MOGNO B, the dose rate is reduced by fourfold from 3.4 to 0.77 mGy/s/mA between the intermediate and higher energy spectra from the focusing mirror for a 7.07 mm Si filter. These energy effects on dose rate distributions across a 2.9-mm soil sample are illustrated in Supplementary Information 1-Figure S4. It is noteworthy that the absorbed dose rate in the root and soil decreases with the increase in monochromatic energy (Supplementary Information 1-Figure S4). This outcome is because the soil absorbs most of the low energy photons before the beam reaches the root (beam hardening effect). Similarly, the zone of higher dose rate behind the root is attributable to the lower absorption coefficient of the root itself. This phenomenon will be inherently less impactful at MOGNO-B using higher energy quasi-monochromatic radiation.

**Table 4:** Modeled X-ray transmission through samples of varying diameter and absorbed dose rates on a root positioned in the center of these samples in relation to X-ray beam energy spectra at MOGNO-A and MOGNO-B and different fields of view (FOVs) as shown in Fig. 3b for MOGNO-B.

	Energy spectrum†	Silicon filter thickness (mm)	Sample diameter (mm)	Sample transmission (%)	FOV (mm)	Absorbed dose rate (mGy/s/mA)
MOGNO-A	Polychromatic	0.36	2.9	50	2.9	8,900 ± 600
		2.45	2.9	70	2.9	3,100 ± 300
MOGNO-B Quasi-monochromatic	Low	0.71	2.9	30 (22 keV)/76 (39 keV)	0.077	55,680 ± 10
					2.9	48.7 ± 0.4
	Intermediate	7.07	2.9	30 (22 keV)/76 (39 keV)	0.077	3,688 ± 3
					2.9	3.4 ± 0.1
			14	< 1 (22 keV)/30 (39 keV)	0.077	1,039 ± 3
					14	0.51 ± 0.02
	High	7.07	2.9	90 (67 keV)	0.077	748.7 ± 0.7
					2.9	0.77 ± 0.03
			40	26 (67 keV)	0.077	230.7 ± 0.9
					40	0.0017 ± 0.0002

^†^Energy spectra are shown in Figs. 1a and 3a.

Our modeling results also indicate that decreasing the FOV to achieve greater spatial resolution on a sample at MOGNO-B increases dose rate by 3- to 5-orders of magnitude for a given sample size and energy (Table 4). This effect is a result of higher flux density on the root when the beam is focused into a smaller FOV. By increasing the sample thickness from 2.9 mm to 14 or 40 mm, as will be possible at MOGNO-B, the modeled dose rate was diminished by at least threefold for all tested FOVs compared with dose rates calculated for the 2.9-mm sample diameter and FOV at MOGNO-A.

## Discussion

Modeling radiation dose largely explained sharply decreasing root growth during XCT imaging with the MOGNO-A beamline configuration, with the incident X-ray energy spectrum, acquisition time as a function of stored current, and acentric position of the root in soil being important factors affecting damage. Dose modeling using the MOGNO-B configuration indicated that dose-induced damage could be decreased by up to six orders of magnitude, depending on the energy and FOV used. Achieving high-quality/high-resolution XCT images and minimizing dose are opposing objectives. Image contrast arises from X-ray interactions with matter that often causes radiation damage to living tissues^23^. However, our modeling results (Table 4) will help users optimize tradeoffs between image spatial resolution, FOV, attenuation, and dose for experiments at MOGNO-B with different analytical objectives. The modelling approach could also be adapted to other transmission XCT beamlines with quasi-parallel (similar to MOGNO-A) or cone (similar to MOGNO-B) beam configurations.

Radiation energy, which depended on Si thickness at MOGNO-A, had an important effect on dose rate (Fig. 5b). Thus, root damage at MOGNO-B, with three quasi-monochromatic energies (Fig. 3a), is expected to be greatly diminished compared with our observations at MOGNO-A (sample diameter and FOV of 2.9 mm; Table 4). This effect is due to the reduction in low-energy flux by optical elements since dose depends on the photon flux and energy. In other words, the quasi-monochromatic energy spectra of MOGNO-B produces a lower amount of energy deposition in matter than the spectra of MOGNO-A.

However, zoom tomography allows imaging roots and soils with different FOVs and spatial resolutions, which constrains the energies used. For example, at MOGNO-B, although the maximum FOV is 85 mm, the sample thickness that can be imaged is limited by the total attenuation (and consequently the transmission) by the composing soil elements and porosity. Thus, it is estimated that for a soil with a typical composition and bulk density of a Brazilian Oxisol, sample thicknesses of 2.9, 14, and ~ 40 mm will transmit ~ 30% of X-rays with lower (22 keV more prominent), intermediate (39 keV more prominent), and higher (67 keV) energy spectra from MOGNO-B, respectively. For XCT image collection of hard samples such as soils and rocks, 30% is considered as an optimal transmission for good quality images. Nevertheless, since this condition is not always possible, a common practice is to use a setup with a transmission that is neither too low (≤ 10%) nor too high (≥ 90%). Therefore, the X-ray transmission through a 2.9 mm thick sample in the intermediate and high energy spectra is less favorable to generate an XCT image (i.e., transmission too high). For this reason, targeting 30% transmission, the low energy spectra would be more suitable to image a 2.9 mm thick sample. However, one should keep in mind that in this particular case, the dose will be critical in the smallest FOV (highest image resolution) (Table 4).

For the setup of higher spatial resolution, which requires smaller distances between the focus and the sample, the energy deposition is concentrated in a smaller FOV and substantially increases the delivered dose relative to the larger FOVs at MOGNO-B for any of the scenarios (Table 4). Hence, greater damage might occur to radiosensitive samples imaged in the region of high spatial resolutions compared to regions of lower ones, although irradiating a much smaller volume of the root might also be a mitigating factor. Besides, as we demonstrated here, the soil can help shield the root from radiation damage (Figs. 5b,c,d). Therefore, the absorbed dose rates for larger sample diameters and smaller FOV at the intermediate and hard energy spectra (MOGNO-B) remains lower than those simulated for the MOGNO-A configuration (Table 4) in which we observed substantial reduction in root growth. Particularly, increasing the soil diameter at MOGNO-B to 40 mm, results in an absorbed dose rate in the 0.077 mm FOV of at least one order of magnitude lower than that estimated for the 2.9 mm sample holder and FOV for the MOGNO-A. For the equivalent sample diameter and FOV (i.e., 40 mm), the difference is around six orders of magnitude lower than MOGNO-A. In other words, if one wants to image roots growing in the smallest possible FOV at MOGNO (highest possible image resolution) and also wishes the root to absorb the smallest dose possible, it is recommended to prepare samples of ~ 40 mm (soil thickness), with roots ideally growing centered, and use the higher energy spectra at MOGNO-B. However, a soil thickness of 14 mm imaged with the intermediate energy spectra in the smallest FOV still provides lower absorbed dose rate than experienced in MOGNO-A.

During an XCT scan, if the root is growing off center, as was the case in our experiments at MOGNO-A, the length of soil transversed by the beam changes during the measurement, thus also changing the absorbed dose rate on the root, as seen in Fig. 5b. However, we had insufficient observations with roots at different positions to assess from our data the precise effects on incremental and cumulative doses. In experiments E1R1 and E2R1, the root rotated from 180° → 0° and 330° → 150° (see Figs. 2c, 4a, and Table 2), respectively, which would be a good contrasting comparison; however, the dose rate changed between these two experiments (Fig. 5b) due to differences in experimental configurations (Table 1). In experiments E3R1 and E3R2 (same sample, different roots), the experimental configurations were the same (Table 1, Fig. 5b), thus allowing a better comparison: the root rotated from 135° → 315° in E3R1 and from 240° → 60° in E3R2 (see Figs. 2c, 4a, and Table 2), and, in accordance, the absorbed dose was 30% lower for E3R1. However, this difference did not seem to affect the root growth rate. In fact, E3R2 showed a higher growth rate than E3R1 between TP1 and TP2, which is likely more related to the natural variability.

In all experiments, the roots tended to grow along or very close to the pipette tip walls, likely due to better aeration. Notwithstanding, the simulations show that the radial distance of root location is also relevant for the dose rate distribution (Figs. 2d and 5c,d). In addition, it was observed that the 90° → 270° rotation results in the lowest absorbed dose rate for all radial soil cylinder increment volumes (Figs. 2d and 5d, Table 2). The best case for this rotation path is when the root is in the outermost incremental soil cylinder (corresponding to the root growing along the wall), but this is not the best case for rhizosphere studies that aim to assess soil-root interactions because of possible wall effects. Therefore, a reasonable recommendation would be to consider samples in which the root is growing along the rotation axis (most inner soil cylinder increment, 0 – 0.29 mm) (Table 2). In this case, the dose rate is close to the best scenario (minimal doses) and depends less on the range of angular rotation (Table 3). Thus, in future experiments where image depends on millimetric capillary diameters, efforts should be made to facilitate root growth near the center of such capillaries instead of along the walls. The possibility to increase the sample diameter and vary the FOV at MOGNO-B makes it easier to have a primary root more centered, but the same considerations should be made for the lateral roots which tend to be off center. However, because root position affected dose rate by a maximum of fivefold in MOGNO-A compared with the four-orders-of-magnitude effect of X-ray energy spectrum options at MOGNO-B, energy selection is more important for root damage than the less controllable position of the root in the soil.

Our modelling results from MOGNO-A showed that the effects of increasing both the Si thickness (higher energies) and the acquisition time (because of the same stored current) from E1R1 to E2R1 compensated each other, leading to similar incremental and cumulative doses (Table 2). In this case, root positioning effects were negligible due to symmetric angular range configurations. On the other hand, with the same Si thickness between E2 and E3, the reduction in acquisition time due to the higher stored current compensated for effects of root positioning and starting angle of the 180° rotation. The stored ring current will be up to 35-fold higher at MOGNO-B compared with MOGNO-A, which will increase the dose rate for a given experimental configuration but will reduce the acquisition time, which was shown to be an important factor influencing the incremental and cumulative dose.

It is noteworthy that the cumulative absorbed dose that was sufficient to reduce the growth rate to ~ 0.05 mm/h in E3R1 and E3R2 (TP4) was similar to the initial (TP1) absorbed dose of E1R1 and E2R1 (Table 2, Fig. 5a). If the absorbed dose was the only factor affecting the reduction in growth rate, we would expect that this initial absorbed dose would also decrease the growth rate to almost zero in the case of E1R1 and E2R1. Notwithstanding, a threefold higher absorbed dose was necessary to almost stop the root growth in E1R1 and E2R1. This result indicates that other factors were influencing the root growth rate. An interesting exemplifying result is that the absorbed dose in E2R2, after only one TP, was very similar to the cumulative absorbed dose in E1R1 and E2R1 after a total of four TPs (Table 2). However, the root continued growing until fully crossing the FOV (Fig. 4c,d,e). This corroborates that the absorbed dose is not the only factor affecting the root growth rate. Thus, we hypothesize that the dose fractionation due to repeated scans might be a relevant factor in this observed behavior.

Blaser et al.^3^ pointed out that a limitation found using the Rad Pro calculator to determine doses was that it did not distinguish between total dose as one single scan or several scans, as we accomplished in the present study. These authors pointed to the concept of “biologically equivalent doses” of different scan frequencies that exists in clinical radiobiology and stated that it is uncertain whether this also applies to root tissue. Our results seem to corroborate that the scan frequency is a relevant factor on root damage, with evidence that it is more relevant than the total absorbed dose itself along an experiment (Table 2; Fig. 4c,d,e). One could argue that the second root (which was probably a lateral one) could be more resistant to dose than the first root, but keep in mind that we presented a counter example in E3R2, which was affected by the dose just as much as E3R1 (two different roots of a same sample). Although we provided some clues to understand this unexpected behavior, more information is needed to explain it thoroughly and predict the implications of the number of exposures to root damage. In addition, although we only measured the reduction in root growth caused by the modeled X-ray dose, there could be other biological effects on the plants that should be studied, such as reductions in the leaf area and shoot weight^3^.

## Conclusions

X-ray dose simulations with the FLUKA model and full accounting for X-ray beam changes along the entire path from the Sirius machine to the root in a soil indicated that diminished root growth observed in our XCT imaging experiments at the MOGNO-A beamline configuration was due to radiation damage. Modeling showed orders of magnitude reductions in dose for the MOGNO-B configuration, mostly due to the higher energies available. Energy, sample thickness, FOV, and ideally root position in the soil sample can all be manipulated to optimize the balance between radiation absorption effects on image quality and radiation damage, consistent with a given experimental objective. Although our simulations were specific to the MOGNO beamline at the Sirius synchrotron, the principles developed in this research with respect to root damage and imaging parameters are more broadly applicable to any X-ray imaging instrument or facility.

## Supplementary Information

Supplementary Information 1.

Supplementary Information 2.

Supplementary Information 3.

Supplementary Information 4.

## Data Availability

The datasets generated during and/or analyzed during the current study are available from the corresponding author on reasonable request.
