# Supplementary material for: Monte Carlo simulations of synchrotron X-ray dose affecting root growth during in vivo tomographic imaging
Source: Sci Rep. 2023 Apr 6;13:5643. doi: 10.1038/s41598-023-32540-5 (PMC10079845; doi:10.1038/s41598-023-32540-5)
Supplement: Supplementary file 1 — Supplementary Information 1. [file 41598_2023_32540_MOESM1_ESM.docx]

**Monte Carlo simulations of synchrotron X-ray dose affecting root growth during *in-vivo* tomographic imaging**

Supplementary information

*Method 1: Soil preparation and wheat seedling germination and planting*

A bulk soil sample was collected from a clayey Oxisol/Red Latosol –(22°45'51.2"S 47°05'29.4"W) at 0 – 20 cm depth. The sample was air dried and sieved to <1 mm through a stainless-steel mesh (Fig. S1a). Pipet tips (100 - 1000 µL, ~15 replicates/batch) were filled with the dry-sieved soil to a level of 10 mm below the top using a spatula, without further mechanical packing (Fig. S1b). Soil-filled pipet tips were arranged in a tip box with holes in the bottom (Fig. S1b) and placed in a plastic bin. Deionized water was slowly added to a height of ~13 mm above the outlets of the tips and left overnight to fully saturate the soil above the XCT imaging FOV between 9.6 and 12.5 mm height (Fig. 1b of the main text) and partially saturate the remaining soil material.

Wheat seeds were germinated, crease down, in a 14-cm diameter, covered acrylic Petri dish on Whatman filter paper saturated with deionized (DI) water, cut slightly smaller than the petri dish to account for swelling. Seedlings with ~3-mm long radicles were planted in the soil filled pipet tips (Figs. S1c,d). For planting, a small hole was made in the center of the wet soil surface using a toothpick to accommodate the seedling radicle. Using a forceps, the pre-selected germinated wheat seedlings were carefully placed in the soil hole so that the radicle pointed downward (as best as possible) in the centered indentation, leaving the hypocotyl and remaining seed on the soil surface (Fig. S1d, 2a). The soil was pushed around the radicle with the toothpick to secure the seedling, and wetted with three drops of DI water to improve soil/radicle contact. The pipet tips were returned to the racks/boxes inside the DI water filled tray (Fig. S1d) and the tray was covered with plastic film. Numerous pinholes were made in the film to aerate while maintaining a higher relative humidity.

The planted seedlings were grown with a 10-hour artificial light/ 14-hour dark (25 ºC) photoperiod for 80 ± 4 hours, a time period estimated from preliminary experiments for roots to grow into the imaging window during XCT data collection. Five samples met these conditions. Wheat samples with similar shoot lengths of 65 ± 10 mm and with no visible roots emerging from the pipet tip were selected for XCT analysis. The bottom of each pipet tip opening was covered with parafilm to prevent water loss. The pipet tips were then mounted in standard sample holders used at the MOGNO-A beamline (Fig. S1e) and fixed with Super Bonder® acrylic glue for mechanical stability during the tomography measurement. Aluminum foil was placed over the top of the pipet tip around the shoot to minimize dehydration during the experiment.


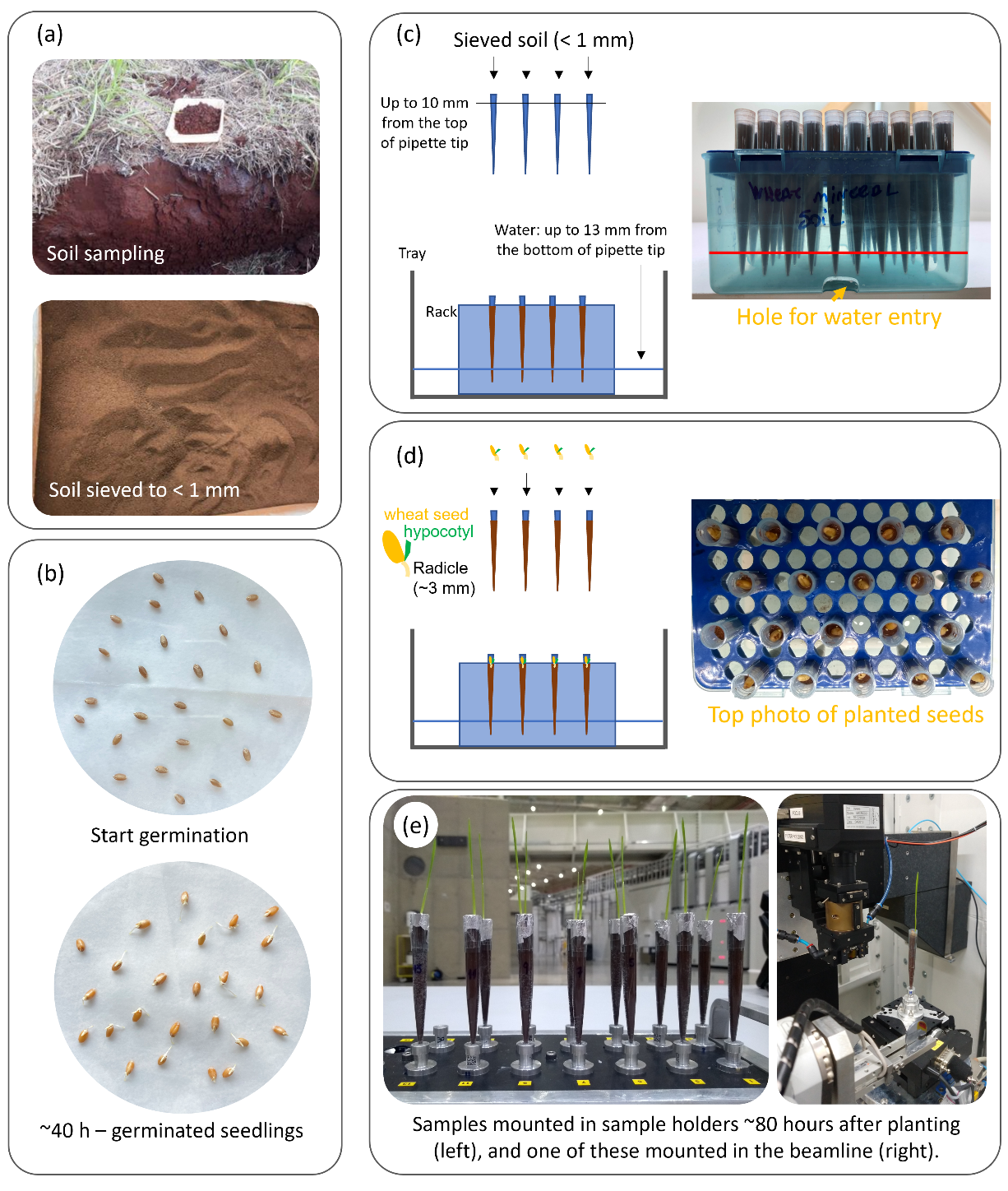


Fig. S1 Schematics showing steps of sample preparation: a) soil sampling and sieving, b) wheat seedling germination, c) preparation and wetting of soil-filled pipet tips, d) planting of germinated seedlings in the pipet tips, and e) mounting of pipette tips in sample holders for SRXCT measurements.

*Method 2: XCT imaging*

X-ray transmission of each experimental unit was defined as the ratio of transmitted to incoming beam (I_t_ / I_0_), where I_t_ was measured on the areal detector at the center of the image projection where the beam passes through the radius of the pipet tip midway down its taper, and I_0_ was the flat field intensity without a sample.

The geometrical resolution (σ_R_) of the reconstructed images was 2.87 × 2.87 µm^2^ (horizontal x vertical), calculated as follows (Bartels, 2013; Krenkel et al., 2015), and was equivalent to the pixel size of the detector ($\sigma_{D}=2.88 \mu m)$:

$\sigma_{R}=\sqrt{\sigma_{R}=\sqrt{\left( 1-\frac{1}{m} \right)^{2}\sigma_{S}^{2}+\frac{1}{m^{2}}\sigma_{D}^{2}}\left( 1-\frac{1}{m} \right)^{2}\sigma_{S}^{2}+\frac{1}{m^{2}}\sigma_{D}^{2}}$ $\sigma_{R}=\sqrt{\left( 1-\frac{1}{m} \right)^{2}\sigma_{S}^{2}+\frac{1}{m^{2}}\sigma_{D}^{2}}$ (S1),

where σ_s_ = is the source size (22.1 × 8.5 µm^2^ – horizontal × vertical); $m is the local magnification=1+\frac{Z_{2}}{Z_{1}}$, where the source-to-sample distance (Z_1_) was 22,915 mm and the sample-to-detector distance (Z_2_) was 117 mm. Note that $\frac{\sigma_{D}}{m}$ is the effective pixel size (σ_eff_). The geometrical resolution will be referred as spatial resolution, although it is known that the final spatial resolution of a 3D image is typically decreased by factors such as noise.

*Figure S2*


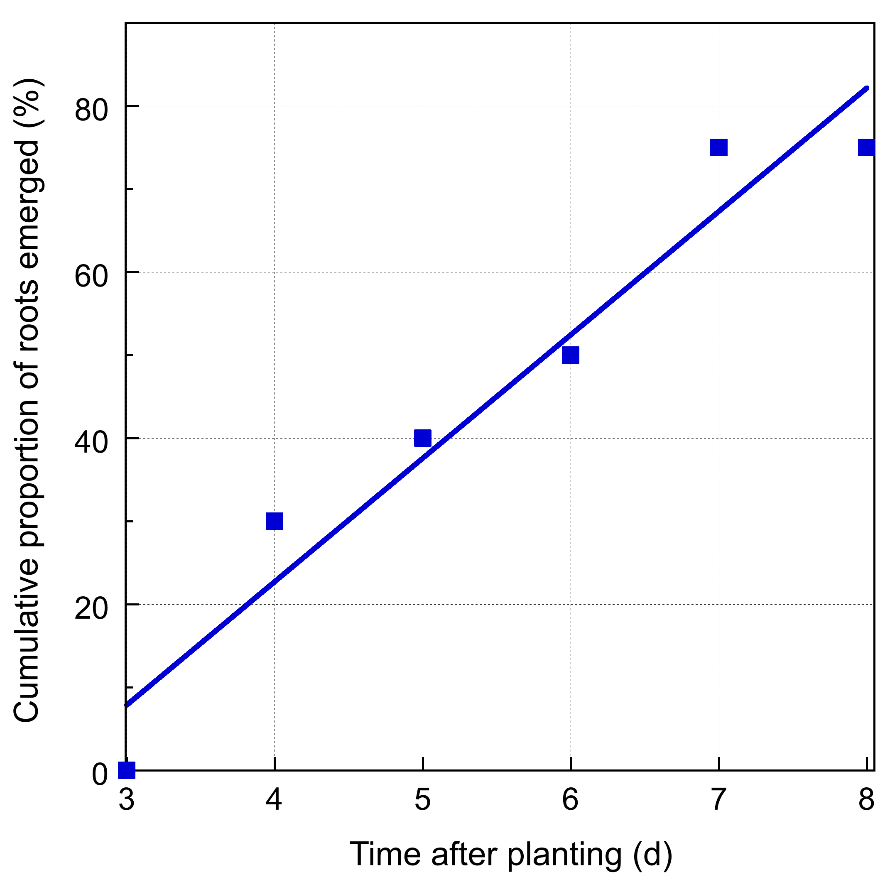


Fig. S2. Results from preliminary experiments showing the trend in root emergence from the 0.7-mm diameter outlet (Fig. 1b, main text) of 100-1000 µL pipet tips in relation to time-after-planting pre-germinated wheat seeds. The linear model fit to the data (y = 36.7 + 14.9 x; R^2^ = 0.94) is only intended to show a trend; no roots emerged from the pipet tips at times ≤3 d.

*Table S1*

Table S1. Composition of air-dried soil based on X-ray fluorescence spectrometry, balanced by oxygen.

| Element | Proportion (% w/w) |
| --- | --- |
| Oxygen | 56 |
| Silicon | 22.3 |
| Aluminum | 12.2 |
| Iron | 5.3 |
| Titanium | 3.0 |
| Calcium | 0.13 |
| Magnesium | 0.064 |
| Potassium | 0.056 |
| Zirconium | 0.032 |
| Zinc | 0.006 |
| Strontium | 0.0028 |

*Figure S3*

*
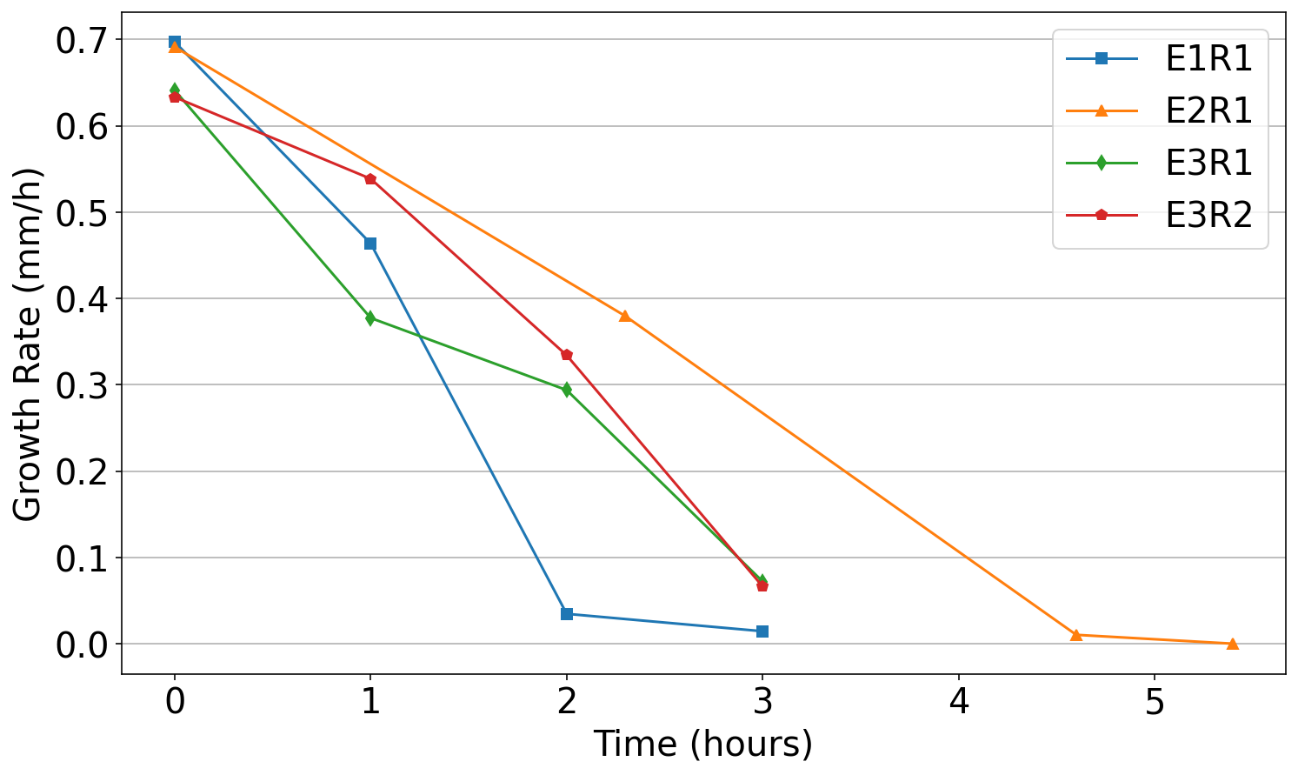
*

Fig. S3. Root growth rate over time for each experiment.

*Figure S4*


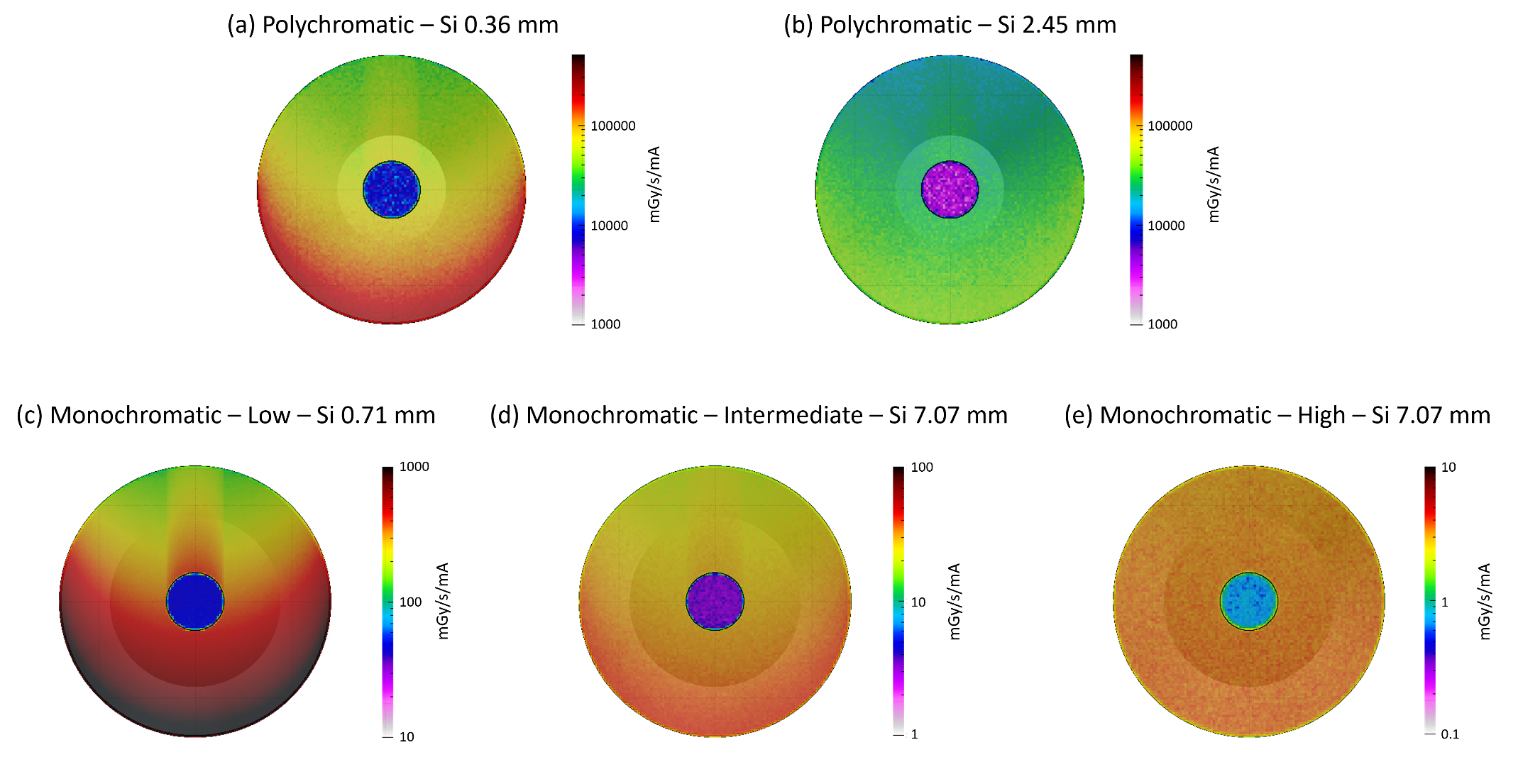


Fig. S4. Modeled dose rate distribution over the soil and root cross sections for polychromatic (a-b) and quasi-monochromatic (c - e) beams for a sample diameter and FOV of 2.9 mm each. Note that the scale bars for dose rates vary by orders of magnitude between figures (c - e).
