# Supplementary material for: Monte Carlo simulations of synchrotron X-ray dose affecting root growth during in vivo tomographic imaging
Source: Sci Rep. 2023 Apr 6;13:5643. doi: 10.1038/s41598-023-32540-5 (PMC10079845; doi:10.1038/s41598-023-32540-5)
Supplement: Supplementary file 4 — Supplementary Information 4. [file 41598_2023_32540_MOESM4_ESM.pptx]

## Slide 1
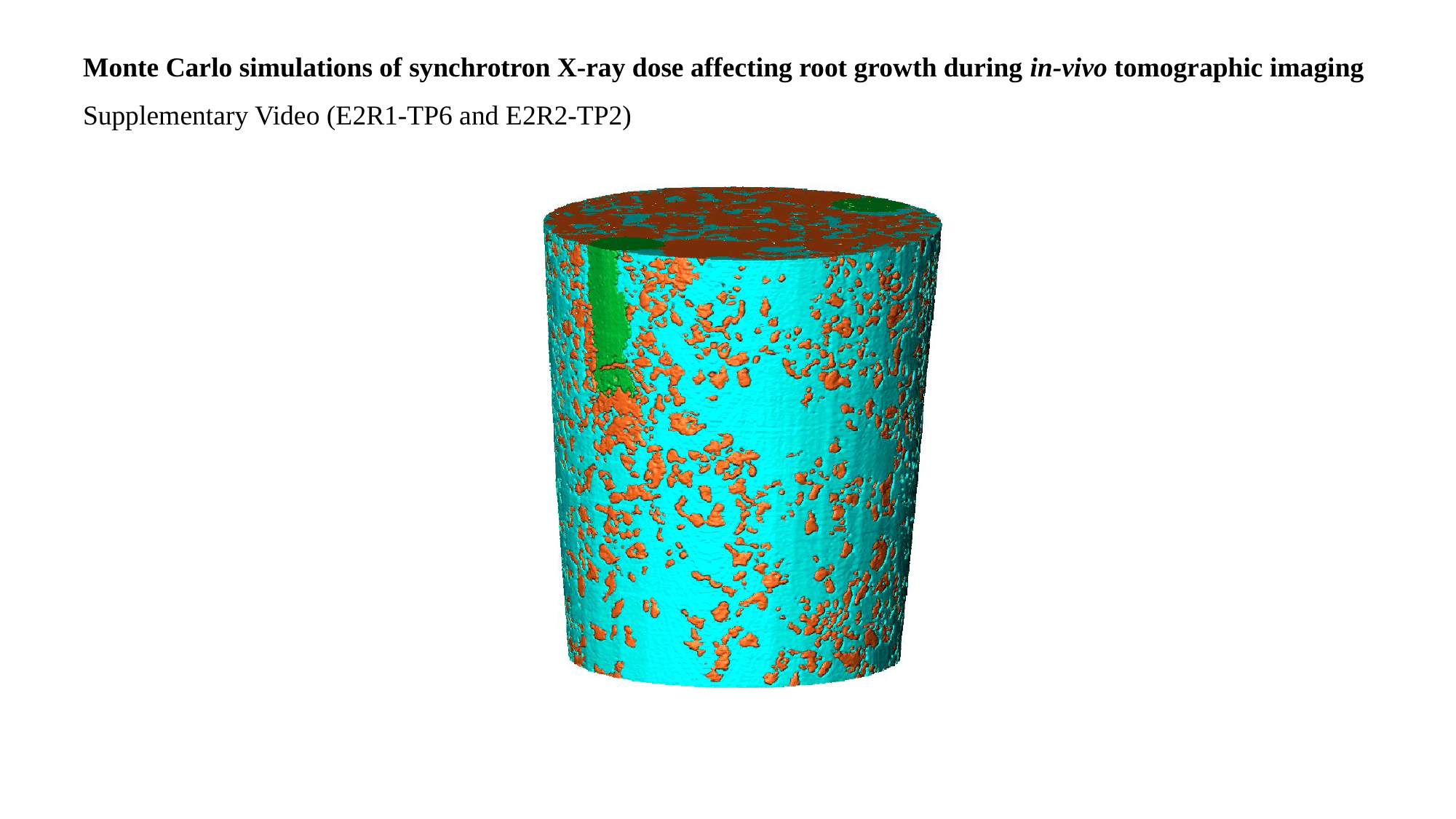

Monte Carlo simulations of synchrotron X-ray dose affecting root growth during in-vivo tomographic imaging
Supplementary Video (E2R1-TP6 and E2R2-TP2)
